# Study protocol of a randomized controlled trial comparing two linkage models for HIV prevention and treatment in justice-involved persons

**DOI:** 10.1186/s12879-022-07354-x

**Published:** 2022-04-15

**Authors:** Sandra A. Springer, Ank E. Nijhawan, Kevin Knight, Irene Kuo, Angela Di Paola, Esther Schlossberg, Cynthia A. Frank, Mark Sanchez, Jennifer Pankow, Randi P. Proffitt, Wayne Lehman, Zoe Pulitzer, Kelly Thompson, Sandra Violette, Kathleen K. Harding, ACTION Cooperative Group, Ralph Brooks, Robert Heimer, Alysse Schultheis, Brent Van der Wyk, Laura Hansen, M. Brynn Torres, Jenny Becan, Ahrein Johnson Bennett, Rachel Crawley, George Joe, Justin Jones, Stephanie Villare, Czarina Behrends, Ali Jalali, Jennifer Muggeo, Melissa Acosta, Dustin DeMoss, Donna Persaud, Jill Johannsen-Love, Frank Davis

**Affiliations:** 1 Department of Internal Medicine, Section of Infectious Disease, Yale School of Medicine, 135 College Street, Suite 323, New Haven, CT 06510 USA; 2 Department of Internal Medicine, Division of Infectious Diseases, Newington VA Medical Center, Newington, CT USA; 3 Department of Internal Medicine, Division of Infectious Diseases and Geographic Medicine, University of Texas Southwestern Medical Center, Austin, TX USA; 4 Institute of Behavioral Research, College of Science and Engineering, Texas Christian University, Fort Worth, TX USA; 5 Department of Epidemiology and Biostatistics, Milken Institute School of Public Health, The George Washington University, Washington, DC USA; 6 Alliance for Living, New London, CT USA; 7 Connecticut Department of Correction, Wethersfield, CT USA; 8 Community Health Center, Inc, Middletown, CT USA

**Keywords:** Mobile health unit, Patient navigator, Opioid use disorder, Stimulant use disorder, Justice involvement, HIV, Hepatitis C, Pre-exposure prophylaxis, PrEP, Sexually transmitted infections

## Abstract

**Background:**

Persons involved in the justice system are at high risk for HIV and drug overdose upon release to the community. This manuscript describes a randomized controlled trial of two evidence-based linkage interventions for provision of HIV prevention and treatment and substance use disorder (SUD) services in four high risk communities to assess which is more effective at addressing these needs upon reentry to the community from the justice system.

**Methods:**

This is a 5-year hybrid type 1 effectiveness-implementation randomized controlled trial that compares two models (Patient Navigation [PN] or Mobile Health Unit [MHU] service delivery) of linking justice-involved individuals to the continuum of community-based HIV and SUD prevention and treatment service cascades of care. A total of 864 justice-involved individuals in four US communities with pre-arrest histories of opioid and/or stimulant use who are living with or at-risk of HIV will be randomized to receive either: (a) PN*,* wherein patient navigators will link study participants to community-based service providers; or (b) services delivered via an MHU*,* wherein study participants will be provided integrated HIV prevention/ treatment services and SUD services. The six-month post-release intervention will focus on access to pre-exposure prophylaxis (PrEP) for those without HIV and antiretroviral treatment (ART) for people living with HIV (PLH). Secondary outcomes will examine the continuum of PrEP and HIV care, including: HIV viral load, PrEP/ ART adherence; HIV risk behaviors; HCV testing and linkage to treatment; and sexually transmitted infection incidence and treatment. Additionally, opioid and other substance use disorder diagnoses, prescription, receipt, and retention on medication for opioid use disorder; opioid and stimulant use; and overdose will also be assessed. Primary implementation outcomes include feasibility, acceptability, sustainability, and costs required to implement and sustain the approaches as well as to scale-up in additional communities.

**Discussion:**

Results from this project will help inform future methods of delivery of prevention, testing, and treatment of HIV, HCV, substance use disorders (particularly for opioids and stimulants), and sexually transmitted infections for justice-involved individuals in the community.

*Trial registration:* Clincialtrials.gov NCT05286879 March 18, 2022.

## Background

Stimulant and opioid use are causing an increasing number of overdose deaths and are also fueling new HIV epidemics in the United States (US) [1–3]. Justice-involved individuals represent an underserved and vulnerable population with higher prevalence of substance use disorders (SUDs), [4] HIV and hepatitis C virus (HCV) than those in the general population [5–7]. A substantial proportion of people living with HIV (PLH) in custody are not engaged in HIV care prior to incarceration and, even when provided care during custody, subsequent engagement and retention in HIV care and viral suppression (VS) drop dramatically after release [8]. For individuals involved in the justice system, there is a unique opportunity to provide access to medications for opioid use disorder (MOUD) [9] and other substance use disorder treatments, as well as to engage them in the full cascade of evidence-based HIV prevention (PrEP) and treatment (antiretroviral therapy, ART) services. Therefore, expedited linkage to integrated services has the potential to substantially reduce the risks of justice-involved individuals acquiring and transmitting HIV and HCV infections while in the community.

The US Department of Health and Human Services developed the *Ending the HIV Epidemic in the US* plan [10, 11] which highlights the need for diagnosing, treating, preventing, and predicting HIV infections. Specifically, this plan includes the prevention strategy of providing PrEP for those at risk for acquiring HIV. However, people involved in the justice system without HIV do not typically access PrEP [12–14]. Furthermore, persons who are prescribed PrEP prior to entering the justice system often lose access to their medication upon incarceration. Evaluation of interventions to improve linkage to and receipt of HIV prevention and treatment services as well as SUD treatments are urgently needed for justice-involved populations. Two models of care, Peer/Patient Navigation (PN) and Mobile Health Units (MHUs), have been shown individually to have some success in engaging persons in substance use and HIV related care; however, there have been few evaluations comparing these models with respect to prevention, testing, and treatment of HIV, HCV, and substance use among justice-involved persons [15].

PN models have emerged as effective interventions to improve retention in HIV care and ART adherence upon release to the community for justice-involved persons [16]. In a large, randomized trial of a structured behavioral intervention administered by a peer navigator among PLH being released from the Los Angeles County Jail, VS was maintained in 49.6% of the PN group compared to 36% of controls who received standard transitional case management alone [17]. Similar outcomes have been reported among PLH receiving care in specialized “transitions clinics” where peer navigators assist released PLH with primary care [18]. PN have been found to be effective in supporting linkage to and engagement in MOUD treatment [19]. Despite this evidence and recommendations by the Centers for Disease Control and Prevention [20], PN services are available in few jurisdictions and community clinics around the US and have not been evaluated for the full HIV, HCV, and SUD care cascades for persons receiving these services.

MHUs have demonstrated the potential to serve as a cost-effective clinical model to deliver healthcare services to underserved populations [21–23]. By being able to be flexibly located and embedded within underserved communities, MHUs’ outreach capabilities include an expanded reach to individuals not typically served by traditional health care systems and can overcome structural barriers to care such as transportation and health system complexity [22, 24]. By offering healthcare in a way that is more patient-centric (i.e., convenient locations, familiar environment, informal setting, and culturally competent staff) [22, 25], MHUs tend to build more trust in relationships with the communities they serve. Study results support the favorable impact MHUs have on improved individual health outcomes as well as population health; they also have the potential to reduce healthcare costs when compared to traditional clinical settings [22]. MHUs can also address both social determinants of health by reaching into underserved communities to overcome structural barriers to care. However, MHUs have not been evaluated with respect to the specific delivery of combined HIV, HCV, and SUD care cascades for justice-involved persons. Therefore, rigorous evaluation of the two models of service delivery for the full range of HIV, SUD (particularly OUD), and HCV care cascades is needed for justice-involved persons. This study will compare PN and MHU provision of care to improve linkage to and engagement in HIV, HCV, and SUD prevention and treatment services in four distinct geographic communities in the US.

## Methods

### Study design and settings

ACTION (Addressing risk through Community Treatment for Infectious disease and Opioid use disorder Now) is a five-year hybrid type 1 effectiveness-implementation randomized control trial which compares noninferiority of two models of linking justice-involved individuals to the continuum of community-based HIV and SUD prevention and treatment service cascades of care funded by the National Institute on Drug Abuse. Adults with a history of opioid and/or stimulant use who are currently incarcerated or were involved with the justice system within the past 30 days will be recruited across two Connecticut communities (New London and Windham/Tolland/Middlesex Counties) and two Texas communities (Dallas and Tarrant Counties). A total of 864 participants will be enrolled in the project, 216 from each of the four project sites. Participants will be randomized 1:1 to one of two models of care: (1) a PN for linkage to care, or (2) services delivered via a MHU for a six-month intervention period in each model. For the PN arm of the study, the navigators will assist in linking study participants to appropriate community service providers. Services include SUD treatment, including MOUD, and HCV testing and treatment. PLH will receive assistance with gaining initial or continued access to antiretroviral therapy (ART) services, and those at risk for HIV will be provided access to PrEP services. For those randomized to the MHU arm of the study, these clinical services will be provided along with the assistance of a community health worker (CHW) on board the MHU. When possible, participants will receive PrEP/ART services and prescriptions, SUD screening and diagnoses, MOUD prescription, and harm reductions services on the MHU. If a service cannot be provided directly on the MHU, participants will be linked to the appropriate community-based service providers. This study is multi-site collaboration with Yale University, the University of Texas Southwestern, and Texas Christian University, along with their community and justice system partners. The study has been funded from September of 2020 through August of 2025.

### Project aims

**Fig. 1:**
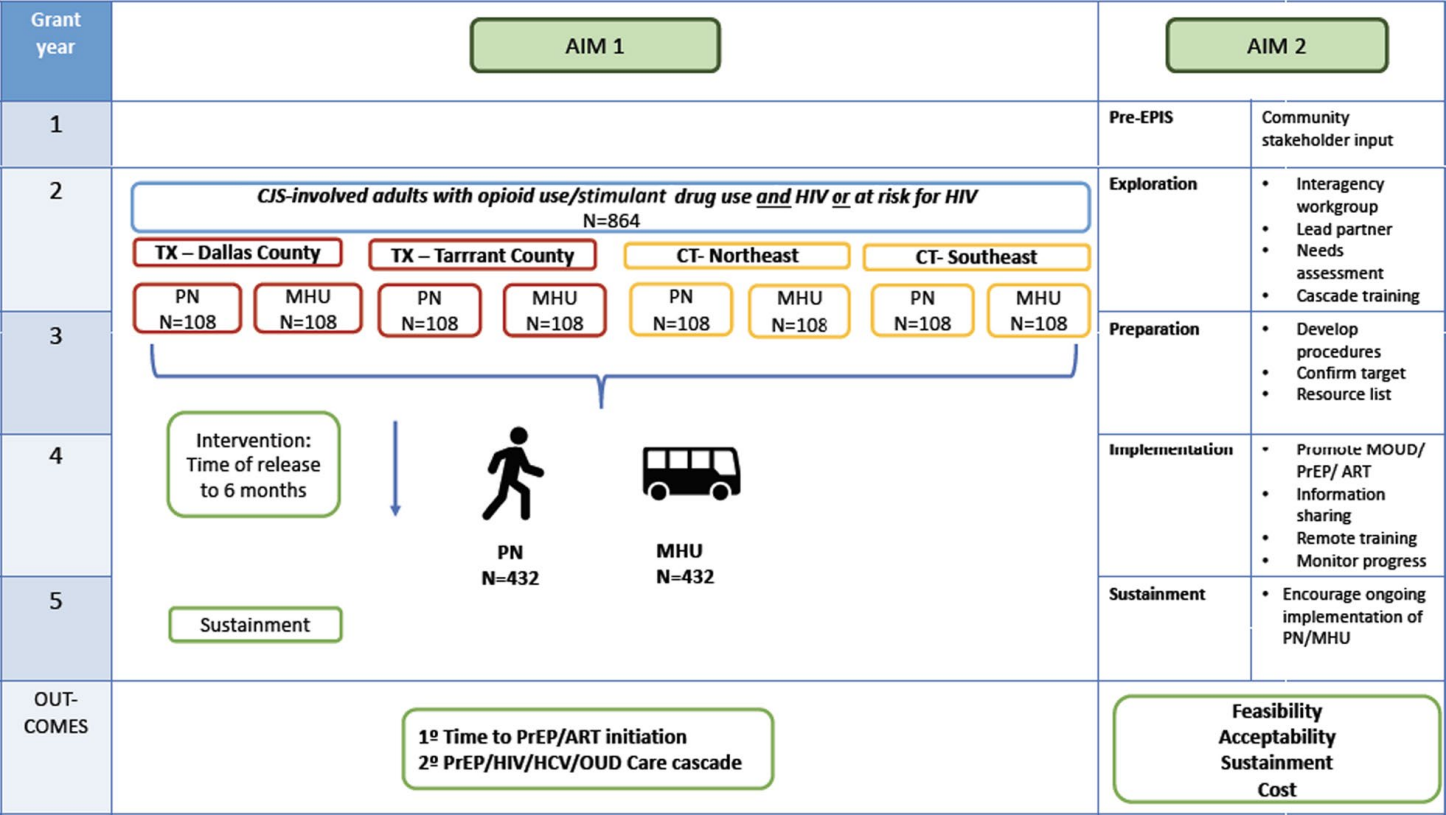
Project design

The primary aim of this study is to compare the effectiveness of PN versus MHU service delivery on participant length of time to initiation of PrEP or ART medication following release from custody, or post-randomization for those enrolled in the community (Fig. 1). Additional outcomes will examine the continuation of PrEP or HIV care, including (but not limited to) viral suppression, PrEP adherence, and HIV risk behaviors. The secondary aim of this study is to evaluate PN and MHU feasibility and acceptability. Additional aims include assessing cost offsets and effectiveness, as well as the effectiveness of outcomes among a sub-study of people who inject substances. Additional outcomes will examine broader community health care impact, including other health services accessed, expanded SUD services, and common barriers (e.g., stigma) to service access across the community provider spectrum.

### Sample size and power calculations

The primary outcome analysis will compare the effectiveness of using either PN or MHU, with the outcome being the length of time it takes for study participants to take their first dose of recommended medications (ART for PLH or PrEP for those at risk for HIV) post randomization. We hypothesize that the MHU arm will be superior to the PN arm, given the convenience and flexibility of the delivery of services. We expect that around 5% of our sample will be PLH and 95% will be at risk for HIV. We also expect 80% retention at the 6-month follow-up assessment. Using SAS PROC Power [26] for comparing two survival curves with log-rank test, we found that 864 recruited participants are needed (evenly distributed across study sites, n = 216 per project site; to achieve a sample size of 689 at 6-month follow-up) under the assumptions of alpha = 0.05 and 80% power to detect a hazard ratio (HR) between 1.25 and 1.30 for main effects of treatment arms. The effect size is based on the literature on PLH referred to ART and people at risk for HIV referred to PrEP. For PLH, 64% of the patients in the treatment condition (designed to facilitate initiation of ART) compared to around 51% of patients in standard care within 10 months [27], yielding hazard ratio of 1.25 comparing the MHU group to the PN group.

For the those at risk for HIV and at high risk of contracting sexually transmitted infections (STIs), the drop-off between PrEP referral to PrEP initiation may be as high as 90% [27–29]. To our knowledge, there are no published studies of PrEP uptake among justice-involved populations, although several papers describe low knowledge of PrEP and moderate levels of interest in taking PrEP among people who inject drugs and justice-involved persons [30–33]. A recent paper indicated that 25% of incarcerated persons reported being interested in taking PrEP after release [30]; given actual uptake generally is lower than reported intention, we assumed that the uptake would be between 10% in the PN group and 20% at best in the MHU group given easier access.

## Study procedures

To achieve the goals of this project parallel study procedures for each aim will be conducted throughout the project period.

### Implementation and needs assessment procedures

The implementation approach is modeled after the Exploration, Preparation, Implementation, Sustainment (EPIS) implementation science framework [33]. EPIS allows for examination of a change process at multiple levels, across time, and through four successive phases: Exploration, assessing system, organization, provider, and client-level factors that explain service gaps and potential barriers/facilitators for change; Preparation, using that awareness to develop plans for enhanced delivery of each approach; Implementation, training of practices to be adopted; and Sustainment, maintaining the use of the PN and MHU approaches. In each phase, EPIS identifies factors relevant to implementation success in the inner context (within the implementing organization\community itself), outer context (external to, but still influential on, the implementing organization\community), and their interactions [34–37].

A planned Needs Assessment Survey (NAS) will be distributed prior to initiating the two intervention arms and will be repeated annually throughout the project period in each of the four communities documenting treatment referral and linkage processes within the participating communities. The online survey inquiries about existing relationships and referral patterns among justice agencies, medical service providers, and community health providers. NAS participants will include representatives from key stakeholder agencies in each community. These agencies will include prisons and jails, community corrections agencies, county or city health departments, substance use treatment agencies, and HIV service agencies. NAS results are critical for helping to inform the details of the two interventions in each of the four communities.

Upon review of the NAS results, focus groups with a subset of the NAS study population from all four communities will be conducted. The purpose of the focus groups is to collect in-depth qualitative data on the current services provided along the continua of care for HIV, SUD, and HCV. Focus group facilitators (i.e., researchers) will utilize semi-structured questions to allow members to discuss barriers to services delivery and uncover gaps in patient care moving through services among local networks, with emphasis on the implementation challenges and potential solutions for PN programs and MHU service provision. The focus group (qualitative) questions will thematically correspond with NAS (quantitative) measures, allowing ease in triangulation [38] to evaluate if these two data sources align on key pieces of information. Thus, findings from the NAS and focus group both will inform the gaps needed to be addressed by both intervention arms.

Audio focus group recordings will be transcribed and qualitatively coded by at least two research staff. Coding fidelity and data reliability will be addressed with inter-coder exercises to achieve coding agreement [39] when applying primary codes in the official transcripts. With ATLAS.ti [40], transcripts will be coded using a deductive approach [41], to identify primary themes and refined secondary themes [39]. For example, the main code, “communication” will have secondary codes (“inter-agency" and “intra-agency “) to explore more nuanced levels of information flow between and within agencies. Through an iterative process of coding and team debriefings, a consensus approach will be used to control coder bias and shared common understanding of phenomena [34, 42].

### Feasibility and acceptability procedures

Primary implementation outcomes include feasibility (health care utilization impact among justice-involved individuals, contributions of interagency workgroup members on primary outcomes); acceptability (participant satisfaction, perceived usefulness); sustainment (continued utilization), and costs required to implement and sustain the approaches and to scale-up in additional communities. Additional outcomes will examine broader community health care impacts including other health services accessed, expanded opioid use disorder (OUD)/SUD services [43], and common barriers (e.g., stigma) to service access across the community provider spectrum. Given the growing concern regarding overdose and injection-related HIV risk behaviors [1, 15, 44–48] an examination of people who inject substances will focus on gaining insight into participant and social context (inner and outer) factors associated with the effectiveness outcomes.

### Randomized controlled trial procedures

#### Recruitment and screening

Recruitment of participants will occur from multiple facilities and settings serving justice-involved individuals. Participants in Dallas and Tarrant counties will primarily be recruited from health care and substance use treatment facilities where persons involved in the justice system receive care. Connecticut-based participants from both project sites will primarily be recruited from the Connecticut Department of Correction prior to release from incarceration in prison and jail. Sites will also recruit from community-based organizations providing services to justice-involved persons who use or used substances and/or are living with or at risk for HIV. Furthermore, adapted snowball sampling will be utilized for community based referrals [49]. After full enrollment in the project, participants will be provided with recruitment vouchers to refer persons in the community they believe may be eligible for the project. Participants will be compensated in the amount of $20 per referral for up to three [3] people who are successfully enrolled in the project. Potential participants will be approached by experienced research staff who received formal training in the administration of all study procedures and interview questionnaires by qualified trainers. Participants who meet preliminary eligibility criteria (including age, ability to speak English and/or Spanish, and current or recent justice involvement) and state clear interest in study participation, will then be invited to voluntarily complete the screening informed consent process with study staff.

#### Eligibility, informed consent, and enrollment processes

Once a participant meets all the pre-screen inclusion criteria and screening informed consent has been completed, the participant will begin the three-part enrollment process. The three parts include: (1) determining study eligibility; (2) providing written informed consent for the full project, which includes the successful completion of the consent questionnaire; and (3) completing all baseline measures (Table 1. Project Assessment and Timeframes). Once the baseline assessments have been completed, the participant will be considered fully enrolled, and will then be randomized. For those in justice settings where biological assessments cannot be completed at the time of the baseline interview, participants will be randomized after the baseline interview assessments have been completed, and biological assessments will be conducted upon release to the community. Enrollment status and study data or results will not be shared with judicial or correctional authorities, unless specifically authorized by the participant.

**Table 1:** Project assessment and timeframes

Study procedures	Intervention period (Day of Release/Randomization-Month 6)				Post-Intervention
	Baseline	Month 1	Month 3	Month 6	Month 12
Activity and biological tests					
Project screening	X				
Consent & randomization	X				
HIV rapid test or viral load based on status	X			X	X
Hepatitis C rapid test or viral load based on status	X			X	X
Sustained preexposure prophylaxis/antiretroviral therapy adherence (dried tenofovir blood spot)				X	
Urine tenofovir			X	X	X
Urine substance use toxicology	X		X	X	X
Testing for sexually transmitted infections (gonorrhea, chlamydia, hepatitis B, and syphilis)	X			X	
Interview assessments					
Demographics	X				
HIV risk behaviors	X		X	X	X
Timeline followback (self-reported daily opioid and stimulant use)	X		X	X	X
Patient Reported Outcome Measurement Information System (PROMIS) for Quality of Life	X		X	X	X
Patient Health Questionnaire (PHQ9; depression)	X		X	X	X
Visual Analog Scales (VAS) for antiretroviral therapy/preexposure prophylaxis/medication for opioid use disorder adherence		X	X	X	X
Texas Christian University (TCU) Drug Screen	X		X	X	X
Engagement in care (prescription refill for preexposure prophylaxis, antiretroviral therapy, medication for opioid use disorder)		X	X	X	X
Overdose and adverse events		X	X	X	X
Craving for opioids and stimulants	X		X	X	X
Incentives*	$50	$50	$50	$75	$75

^*^Participants will also receive payment of $5 for monthly check-ins on months where no research interviews are being conducted

#### Eligibility criteria

Inclusion criteria are as follows:

(1) Age 18 or older; (2) able to provide written informed consent in English or Spanish; (3) currently in a justice supervised facility (e.g., jail, prison, treatment center, or halfway house) with pending release date within 30 days or involvement with the justice system within the last 30 days (e.g., arrest, probation, parole, or incarceration); (4) willing to have HIV testing to determine status, or confirmed to be living with HIV by a medical chart review; (5) having a pre-custody history of opioid and/or stimulant use within 12 months prior to incarceration; (6) having a pre-custody history of condomless sexual intercourse and/or injection substance use within six months prior to custody; and (7) be willing to learn about PrEP if not living with HIV.

Exclusion criteria are as follows:

(1) Not remaining in the local area after release from custody; (2) having a severe medical or psychiatric disorder making participation unsafe; (3) being released to inpatient care facility; and (4) exhibiting behavior that may have a potential risk to research staff.

#### Post enrollment

Upon completion of the enrollment process, including screening, written informed consent, baseline assessment, and randomization, participants will undergo assessments at months 1, 3, 5 and 12. The following biological tests will be completed at baseline, and months 6 and 12: HIV rapid test or viral load based on status, HCV rapid test or viral load based on status, and testing for STIs (gonorrhea, chlamydia, hepatitis B, and syphilis). To assess for adherence to tenofovir based ART or PrEP medications, urine and a dried blood spot (DBS) will be analyzed at months 3, 6 and 12. Urine toxicology screens will be conducted to assess substance use at baseline and months 3, 6, and 12. Items in the follow-up interview assessments will include: demographic characteristics, sexual and injection HIV risk behaviors, daily opioid and stimulant use and route via the timeline followback, engagement in medical, substance use and social services or treatments, quality of life and pain via the Patient Reported Outcome Measurement Information System (PROMIS), depression symptoms using the Patient Health Questionnaire (PHQ9), TCU Drug Screen 5, craving for opioids and stimulants, and overdose experiences. At each follow-up study visit participants will be asked to complete a medical release of information for community-based providers to allow project staff to verify attendance of medical visits as well as prescription receipt.

#### Participant tracking

At each study visit participants will be asked to provide extensive locator information including a mailing address, phone number, social media contact information, hang out locations, and place of work. If we are not able to contact the participant directly, participants are also asked to provide contact information for a friend or family member as an alternative contact. Project staff will use this information for appointment reminders and to maintain contact with the participant between appointments. If a participant is not able to be contacted project staff will conduct community outreach to the addresses and locations provided. If participants are re-incarcerated during their time in the study, IRB approval and agreements with local correctional facilities have been obtained to allow research staff to follow and interview participants while incarcerated.

## Covariate and outcome measures

**Fig. 2:**
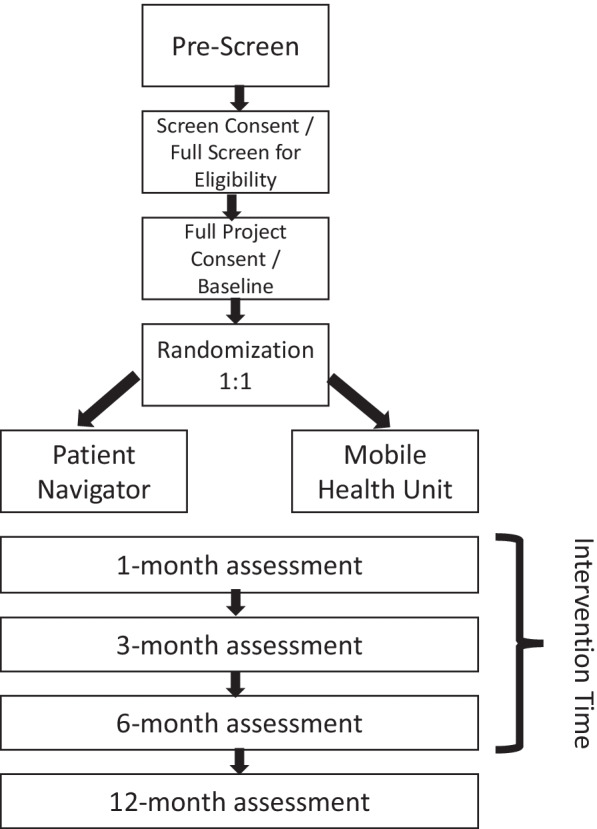
Participant flow through project

Measures used to assess eligibility and intervention effectiveness are listed in Table 1 and described below. There will be a total of five research-related visits conducted in this project, including at the time of baseline and enrollment, during the intervention period at months 1, 3 and 6 post-release/enrollment, and the final visit 6 months post-intervention (12 months post-release/enrollment; Fig. 2). Interview based data will be collected using REDCap interview software.

### Demographic, medical diagnosis, and treatment history

At the time of the baseline assessment demographic characteristics including age, gender, race, ethnicity, housing status, income, and education will be collected. Participants will also be asked about their pre-justice involvement behavior and medical history. Self-reported medical history includes previous diagnosis and treatment of HIV, HCV, sexually transmitted infections (STIs), and other medical diagnoses. HIV and HCV diagnoses will be confirmed through medical chart request. Follow-up assessments include items to assess the change in housing status, income, justice involvement, and new medical diagnoses.

### Healthcare initiation, adherence, and retention of care

Data will be collected to create treatment cascades associated with HIV, PrEP, HCV, MOUD, and STI testing, diagnoses, treatment, and care. Diagnoses and sero-conversion of HIV, HCV, and STIs will be assessed through medical history at every visit and via biological tests noted below. Based on diagnoses and current medications, time to linkage, initiation, or continuation of ART, PrEP, HCV, MOUD, or STI care and medications will be assessed from time of release from justice-based facilities, after randomization for those in the community, or upon diagnosis throughout the 12-month study participation period. Adherence to ART, PrEP, HCV, and STI treatments will be assessed using a visual analog scale (VAS [50]) for daily medications. Biological measures described below will assist with the evaluation of ART and PrEP adherence. For PLH at time of randomization the percent who maintain or achieve HIV viral load < 200 copies/mL at 6 months [51, 52] will be collected. Persistence on medication will be determined by assessing if medication was discontinued and reason for discontinuation, including successful cure of the infection if applicable. Attendance to medical visits will be confirmed with community providers and the MHU.

### Opioid and other substance use

The TCU drug screener [53] will be used to assess for opioid, stimulant, cannabis, synthetic cannabinoid, synthetic cathinone, hallucinogen, inhalant, and benzodiazepine use disorders at baseline, and months 6 and 12. History of substance use related treatment, including use of MOUDs, will be assessed at baseline. At each of the follow-up assessment visits, participants will be asked about current substance use and treatment adherence. Daily opioid and stimulant use, as well as route of administration, will be assessed using the timeline followback [54, 55]. At baseline, the Timeline Followback will ask about opioid and stimulant use for 30 days prior to justice involvement. Follow-up assessments will capture daily use throughout the intervention phase, and the last 30 days at the 12-month visit. Time to first opioid or stimulant use, number of days stimulants and/or opioids were used, and percent days abstinent will be assessed. Participants will also be asked about their experiences with opioid-related overdoses and naloxone use throughout the study period. Mortality and cause of death among participants will be tracked using the Center for Disease Control and Prevention National Death Index request system [56].

### Mental health, stigma, social functioning, pain, and quality of life

Pre-justice involvement mental health diagnoses will be collected. Current depressive symptoms will be assessed using the PHQ9 [57, 58] at baseline and months 3, 6, and 12. Social functioning, quality of life, and pain will be assessed using the PROMIS [59] at baseline and month 3, 6, and 12 visits. Stigma associated with being a person who uses or used substances will be assessed using a harmonized scale from the HIV Prevention Trials Network INTEGRA initiative (HPTN 094) at the same time points.

### Sexual and injection HIV risk behaviors

HIV risk behaviors will be assessed at baseline and follow-up research visits. Sexual and injection substance use behaviors will be assessed using the HIV Risk Behavior tool developed [60] for the National Institute on Drug Abuse’s Seek, Test, Treat, and Retain initiative [60].

### Biological measures

Biological assessments will be conducted throughout the project. These assessments include the following: (1) Rapid HIV testing will be done at baseline, at the end of the intervention (month 6), and at the end of the project (month 12) for those who are not living with HIV. For PLH, HIV viral load (VL) will be obtained from Quest Diagnostics or medical chart records at the same time points; (2) Rapid HCV testing will also be carried out at the baseline visit, those living with HCV will have HCV VL obtained from Quest Diagnostics at the time of release. Repeat HCV testing and VL will be done based on HCV status at months 6 and 12 post-release/enrollment. (3) Urine toxicology assessments for opioids, stimulants, and other substances (specific substances include: amphetamines, barbiturates, benzodiazepines, buprenorphine, cocaine, fentanyl, methadone, methamphetamine, opiates, oxycodone, and phencyclidine) will be done at baseline and months 3, 6 and 12 follow-up visits. (4) DBS and urine specimens will be analyzed to assess for adherence levels of tenofovir based PrEP and ART medications at months 3, 6, and 12 follow-up visits [61, 62]. (5) Gonorrhea, chlamydia, hepatitis B, and syphilis infections will be assessed at baseline and month 6 follow-up visits through urine or DBS analyses.

## Randomization

After eligibility has been determined and informed consent has been obtained, the participant will undergo the baseline assessments and biological tests outlined above in Table 1. Once these have been completed, the participant will be randomized by a research staff member. Simple randomization stratified by project site will be allocated via the REDCap randomization module. Allocation tables were developed by the project Statistician using computer based random number generation. Participants will be assigned to one of the two following groups: Group A, participants assigned to this group will receive help from a PN who will connect them to treatment providers in their local community and Group B, participants assigned to this group will receive care from a project-supported MHU. The participant will be told immediately if they are randomized to receive PN or MHU services.

## Interventions

### Patient navigation (PN) arm

Our PNs will be individuals that ideally have “shared living experiences” (e.g., HIV or history of SUD treatment) with participants and will help them overcome barriers to accessing and engaging in quality care [15]. We will work with our community partners to assure that our PNs are integrated as members of the community health care teams. The PN will work closely with the study participants to access medication for opioid use disorders, behavioral health for stimulant use and other SUDs, HIV and HCV testing, prevention, and treatment, as well as other primary health care. They will also assist with housing, health insurance, transportation, childcare, and other needs during the six-month intervention. Across all four community sites, ACTION PNs will receive active, comprehensive training and guidance on providing appropriate linkage to community services including harm reduction services such as syringe services programs in Connecticut (not available in Texas) and education on safe injection practices of substances to reduce HIV and HCV infection and reinfection.

The community partners collaborating on this project have extensive experience in PN support services, their PN protocols will provide a guiding framework for the ACTION training protocol. PN training will capitalize on existing linkages to an array of services through Federally Qualified Health Centers or similar organizations, including primary care, substance use treatment, behavioral health, case management, and care coordination. In addition, these services include confidential rapid HIV and HCV testing, education about prevention of HIV and HCV care, PrEP, post-exposure prophylaxis for HIV, and MOUD (buprenorphine and extended-release naltrexone). All PNs will be trained to provide motivational interviewing and goal setting as part of their services provided in the project as it has been shown to improve treatment engagement in persons who use substances [63, 64].

### Mobile health unit (MHU) arm

MHUs in the four project areas will either operate and provide care directly on the MHU. All MHUs will provide on-site integrated HIV, HCV, and substance use testing, prevention, diagnosis, treatment, and care. When medications are not able to be provided on the MHU, a clinician on the MHU (or via referral) will provide prescriptions for any medications related to their care, including MOUD (buprenorphine/naltrexone and extended-release naltrexone). Furthermore, the MHU team will have an established relationship with Ryan White Qualified Healthcare programs and other community organizations for linkage to services in the community they are not able to provide directly. A community health worker (CHW) will be on the MHU and will assist in referring and linking participants to community-based services as needed.

Prior to the end of the six-month intervention period, study staff will work to transition participants randomized to the MHU arm to either another MHU that is convenient to them or other community-based services in order to maintain continuity of care. Protocols have been developed in each of the communities in Connecticut and Texas to identify community locations in which to locate the MHUs that will be accessible by the target populations.

## Compensation

Participants will be compensated for their time completing research-related study visits. Upon completion of the baseline assessment (including biological testing) participants will be compensated $50. Follow-up visits conducted 1- and 3-months post-release/enrollment will be compensated at a rate of $50 per completed research visit. Research visits at month 6- and 12- post-release/enrollment will be compensated at $75 per completed research visit. To increase retention, a payment of $5 will be given if the participant is successfully contacted during the months without scheduled follow-up visits which will be conducted to optimize retention. All payments will be in the form of gift cards. To reduce the risk of coercion among those involved in the justice system payment will be given to the participant upon re-entry to the community for those who undergo a research visit in a justice facility.

## Collection and reporting of adverse events

Any unfavorable and unintended sign (including an abnormal laboratory finding), symptom or disease temporally associated with the use of a medical treatment or procedure, regardless of whether it is considered related to the medical treatment or procedure will be noted as an adverse event. All project staff will be trained to recognize, document, and report adverse events. Each adverse event will be classified by the PIs as serious or non-serious and classify each adverse event according to the attribution categories. Each of these adverse events will be graded as (1) mild, an experience that is transient, and requires no special treatment or intervention; (2) moderate, an experience that is alleviated with simple therapeutic treatments, an experience impacts usual daily activities. Or (3) severe, an experience that requires therapeutic treatments or hospitalization. Serious adverse events include events that result in death, serious injury, a life-threatening experience, inpatient hospitalization, persistent or significant disability/incapacity, termination of employment, or institutionalization in residential treatment, hospital, or jail. Adverse events will also be assessed for attribution of the project as (1) not related, the adverse event is clearly not related to the study procedure; (2) possibility related, an event that follows a reasonable temporal sequence from the initiation of study procedures, but that could readily have been produced by other factors; and (3) related, adverse event it clearly related to the project. Any event or outcome that was not described as a risk of participation in the research or, even though described as a risk, which has occurred with unexpected severity or frequency will also be monitored and systematically assessed.

In the event that a participant either withdraws from the study or the investigators decide to discontinue a participant due to a serious adverse event, the participant will be monitored by the PIs via ongoing status assessment until (1) a resolution is reached (e.g., the problem requiring hospitalization has resolved or stabilized with no further changes expected); (2) the serious adverse event is determined to be clearly unrelated to the study intervention; or (3) the serious adverse event results in death.

Adverse events expected to be deemed serious will be reported to the Yale Contact PI (S. Springer) within 24 h of detection. If deemed serious, the PI will send a full report on the serious adverse event, including the action taken with regard to continued study participation to the sIRB and DSMB within 3 business days. Non-serious adverse events that lead to participant removal from the protocol will be reported to the sIRB and DSMB within 7 business days. The PI will maintain a record of the serious adverse event, which can be provided to the sIRB electronically. The outcome of serious adverse events also will be reported to National Institute on Drug Abuse in annual progress reports.

## Analytic plan

### HIV, HCV, and OUD cascades of care

The primary outcome of this project is to assess the difference in time to initiation of PrEP or ART between PN and MHU groups

PrEP initiation will be measured as the date of self-reported initiation within the 6-month intervention period as has been used in other studies of PrEP uptake [65]. Self-reported initiation will be triangulated with prescribing data from the medical record and pharmacy fill date; the same approach will be used to assess post-release ART re-initiation/continuation. If participants are unable to be contacted, when appropriate, medical visit appointment attendance with community providers will be assessed. We will continue to follow individuals if they are returned to custody (with statistical models adjusting for incarceration time, as appropriate).

Time to post-randomization initiation of PrEP/ART will be analyzed as intention-to-treat analyses including all persons fully enrolled in the project using Kaplan–Meier estimates and adjusted Cox proportional hazards models, adjusted for relevant covariates. The effect of treatment on the outcome will be described using the estimated coefficients of the treatment term in the model, the corresponding hazards ratio, and 95% confidence interval. Kaplan–Meier estimates will be used to generate and plot survival curves over time in the PN and MHU groups. The inferences from analyses with missing data are valid provided that they are “missing at random” [66]. ‘Missing at random’ (i.e., does not depend on the value of the unobserved outcome) is un-testable in most medical research and in our study as well. Sensitivity analysis will assess the effect of the assumption of missing ‘at random’ on the inference.

PrEP Cascade-based outcomes will be evaluated as the proportion of participants who initiate PrEP and are on PrEP at end of intervention [67]. These will be calculated as overall percentages and stratified by study arm. Evidence of PrEP adherence will be assessed by DBS testing and defined as a tenofovir-disoproxil diphosphate (TFV-DP) level > 700 fmol/punch [61] at 6 months, reflecting cumulative dosing over 6–8 weeks and consistent with 4 or more doses of PrEP per week [62]. This threshold has been commonly used as the level required to achieve adequate HIV protection, however, as the level of adherence that is required for HIV protection among people who inject drugs is less clear, and there are limited data on measures of PrEP adherence among people who inject drugs [62, 68]. We will triangulate multiple adherence measures: TFV-DP DBS levels as a continuous outcome; point of care TFV-DP urine testing, which measures recent (past 48 h) dosing [69], self-reported PrEP adherence via VAS [50], and pharmacy refill data. The proportion of those tested for HIV with negative results using rapid point of care testing over time will be calculated.

The HIV care cascade will include: HIV incidence for those who seroconvert during the course of the study; post-incarceration ART initiation and adherence: ART initiation (or continuation) post-release defined as time to self-reported initiation of ART after release from the justice system. ART initiation will be triangulated using prescription date data from the community-based medical record (either via PN referral or via MHU visit) and prescription fill date for ARTs; ART adherence is defined as the proportion who have an ART prescription and who are retained on ART, which will be assessed via self-report as well as DBS testing for TDF- and TAF-based regimens; Retention in HIV ART care is defined as attending 2 or more visits in 6-months post-enrollment and will be assessed via self-report; HIV viral suppression will be assessed as the proportion who maintain or achieve viral suppression defined as HIV viral load (VL) < 200 copies/mL at 6 months [8, 10]; HIV Risk Behaviors will be assessed for shared of injection equipment, as well as other substance use, and sexual risk behaviors over time. The HIV care cascade will be calculated overall and stratified by arm.

HCV cascade of care cascade will be assessed as the proportion who have a reactive rapid point of care HCV test with confirmatory reflex HCV VL; the time to linkage for and HCV treatment appointment, and time to receiving HCV direct acting antiviral treatment during the 6 month intervention; the proportion eligible who complete treatment; of those, the proportion who achieve sustained virologic suppression at week 12 (undetectable HCV VL); and of those who achieve sustained virologic suppression [70, 71], the proportion who have HCV re-infection at 12 months. The HCV care cascade will be calculated overall and stratified by arm.

OUD/SUD outcomes and cascades of care will include the proportion who have a current or past diagnosis of SUD (including stimulant and opioid use) by (a) proportion of monthly negative versus positive/missing urine samples, (b) self-reported days of opioid and injection drug use using the timeline followback and other drugs and alcohol using the TCU Drug Screen 5 and Opioid Supplement [53], and c) and percent of opioid and/or stimulant-free months (0–6). In addition, we will track non-fatal and fatal overdose events, addiction treatment participation, and linkage to and retention on MOUD using a brief version of the Treatment Services Review [72] that records type and dose of MOUD and behavioral health. These cascades will be calculated overall and stratified by arm.

For repeated (longitudinal) continuous or categorical outcomes such as adherence and retention in care, longitudinal generalized mixed effect models (MEM) will be fit. The MEMs allow adjustment for potential unequal or time-varying variances. Different link functions will be used depending on each outcome variable: logit-link for binary and categorical outcomes, log-link function for right-skewed distributions, and identity-link function for normally distributed outcomes. The generalized estimation equation structure will be added to model within-subject correlations over time as an autoregressive AR [1] process. Each model will include the main effect of the predictor of interest, main effect of time (baseline, months 1, 3, 6, and 12), their interaction (if the predictor is observed and desired to analyze over time), and relevant covariates (if appropriate). Time to PrEP/ART uptake will be analyzed either as a continuous or categorical predictor if changes over time are not assumed to be linear. Associations of predictors with secondary continuous or categorical outcomes that are not longitudinal will be analyzed using generalized linear models fit similarly as above. However, each model will include the main effect of the predictor of interest and relevant covariates only.

Moderation statistical models will be used to determine the effect of randomization to PN versus MHU arms among subpopulations of the sample based on the demographic and baseline characteristics selected as moderators. These characteristics include age, gender, primary substance used, race, ethnicity, and living in an urban or rural setting. We will estimate the moderation effect on primary outcome using similar models noted above with the inclusion of moderator by treatment interactions in the Cox proportional hazards model. Regardless of the significance of the interaction term, we will use the model to estimate the 95% confidence interval of the treatment effect for each level or category of the moderator variable, or the treatment effect at selected values of the moderator variable, if continuous. The moderator analyses are exploratory and hypothesis generating, but significant moderator effects can be useful in planning future efforts at implementation linkage to and receipt of HIV prevention and treatment services for persons who use substances and are at risk of acquiring or are living with HIV released to the community from the justice system. By identifying subgroups who receive less benefit, modifications to the implementation strategy can be performed to achieve better effects in those subgroups, therefore improving patient care.

### Implementation, feasibility, and adaptation

We will examine changes across time of feasibility, acceptability, adoption, and sustainment for both interventions. The Proc Mixed random intercepts multi-level repeated measures model will be used where variations in changes are estimated and the effects of time, study arm (PN vs. MHU), and their interaction will be tested as fixed effects. Baseline measures of each dependent measure will be used in the analyses as covariates.

Convergence of the data will bolster validity in the findings by merging qualitative and quantitative data to provide insight into the effectiveness of the interventions. This is especially important to promote validity for implementation science studies that typically use nested designs with a small sample of communities [73]. We will (1) assess intra-cluster (community) correlations (all conditions); (2) summarize (descriptive stats) individual and community characteristics for each intervention arm (numbers analyzed, the average cluster size, cluster characteristics, and important staff/client characteristics). If differences exist between conditions, the variables will be included as selected covariates in analytic models. An intention-to-treat approach will be used whereby observations are analyzed in their intended condition. Also, protocol implementation measures will be recorded, summarized, and reviewed monthly in order to inform real-time adjustments (e.g., changes in protocols if adherence to the protocol is not met). At project end, difference in proportions will be calculated as a measure of effect, and large-sample 95% confidence limits calculated as a measure of precision.

### Cost effectiveness

Cost analysis will be conducted to estimate costs of the PN and MHU interventions using established micro-costing methods to identify, measure, and value all resources for the implementation of both interventions. We will estimate costs for the PN and MHU study sites including start-up costs (e.g., staff training, equipment purchases); variable costs (e.g., labor time for staff providing services to clients); time-dependent costs (e.g., record-keeping, vehicle operating costs); and overhead costs. Costing data will be collected by using site records on numbers of services provided to clients, estimates of the time required to provide services obtained through staff interviews, and site financial records. Costs will be calculated by applying national labor rates (to improve generalizability) to staff time estimates or obtaining direct costs of equipment from financial records. Results will be reported as annual costs for each of the arms in total, by cost component (i.e., start-up, ongoing costs, overhead), and per client served. To determine additional healthcare system and societal costs from PN and MHU use, we will use self-reported data on the use of medical and substance use treatment services, criminal activity, and engagement with the justice system outside of the study interventions. This data will be collected from participants in the baseline and follow-up assessments. We will use Medicare fee-for-service payment estimates to approximate healthcare system costs incurred by providers. Differences in levels of health service utilization will be estimated to determine the impact of different interventions on costs to the healthcare system 6 and 12 months after baseline.

All cost analyses will be conducted using a multivariable generalized linear mixed model (GLMM). The GLMM is an extension of the generalized linear model that allows for the inclusion of random effects for each study site. Separate multivariable GLMM will be estimated to predict the mean value for each resource and outcome, at each time period, by model (PN and MHU), and participating community. To account for sampling uncertainty in point estimates, the p-values and standard errors will be estimated using nonparametric bootstrapping techniques. Discounting for time preference will not be required since measures will not be obtained beyond 12 months after the baseline interview.

#### Data management and quality control

A REDCap web-based data system and database will be housed at the Yale University site. To ensure protection of confidentiality, assessment data will be entered into the study database with no personal identifiers and source documents will only identify participants by study ID number. Only authorized field personnel and the Data Manager will have access to the data through the REDCap interface, and direct access to the REDCap backend database is not permitted for any project staff. Access to the data follows the FDA guidelines (21 CFR Part 11) for data protection, including role-based access to study data. The Statistician and Investigators will have access to REDCap’s report generator but not to patient-level data.

The Yale site will lead and coordinate quality assurance monitoring, protocol adherence quality, and assurance in consultation with site Study Coordinators. Adherence to Protocol and Standard Operating Procedures will be monitored through a weekly conference call with the Site Study Coordinators and research staffs, and study monitoring. Furthermore, a Yale researcher, independent of this project trained in quality management, will conduct data quality assurance audits at each study site every six months, with the initial audit conducted upon the enrollment of the first 25 participants randomized at the site. These audits will include reviewing regulatory documents, informed consent forms, documentation of primary outcome data, documentation of adverse events, and protocol deviations.

#### Data safety monitoring board

A Data Safety and Monitoring Board (DSMB) will be assembled in order to monitor adverse events that could occur during the research. Members of the DSMB will include at minimum a representative from criminal justice, a health service provider, a behavioral health provider, and a biostatistician. The DSMB will meet initially before the start of the trial to discuss the protocol, approve the commencement of the trial, and to establish guidelines to monitor the study. A Chair of the Board will be selected at the initial meeting. Meetings of the DSMB will be held two annually at the call of the Chair. An emergency meeting of the DSMB may be called at any time by the Chair should questions of client safety arise. All meetings shall be closed to the public because discussions may address confidential patient data. The DSMB meetings will consist of an open and a closed session. The open sessions may be attended by the PIs and other study staff but should always include the study biostatisticians. Issues discussed at open sessions will include conduct and progress of the study, including client accrual and problems encountered. Participant-specific data and treatment group data may not be presented in the open session. The closed session will be attended only by voting DSMB members and administrative support staff not otherwise affiliated with the study. Others may be requested to attend by the DSMB (e.g., study statistician). All safety and efficacy data are and must be presented at this session. The discussion at the closed session is completely confidential. Should the DSMB decide to issue a termination recommendation, full vote of the DSMB will be required. In the event of a split vote, majority vote will rule and a minority report should be appended.

Interim reports will be prepared by the study statisticians and distributed to the DSMB prior to a meeting. The contents of the report will be determined by the DSMB. Interim data reports will consist of an Open Session Report which provides information on study aspects such as accrual, baseline characteristics, and other general information on study status, and a Closed Session Report which will contain data on study outcomes, including safety data and depending on the study, perhaps efficacy data. The Closed Session Report will be considered confidential. A formal report from the Chair will forward the approved minutes to the PIs with a recommendation to continue or to terminate the study. This recommendation should be made by formal majority vote. On a scheduled basis (as agreed upon by the DSMB) blinded safety data should be communicated to all DSMB members or to the designated safety officer. Any concerns noted should be brought to the attention of the Chair or designated safety officer.

#### Dissemination plan

A dissemination board was created to monitor and track dissemination products from this project. The dissemination board consists of the project PIs and a key co-Investigator. Findings from this project will be presented at national medical and research conferences, as well as via published manuscripts with open access to the public. Findings of interest to the local providers and justice providers will be presented at community level meetings. Authorship will be determined based on contribution to the dissemination product, and policies of the journal or conference.

## Discussion

To our knowledge, ACTION is the first head-to-head comparison of Patient Navigation and Mobile Health Unit linkage interventions to support access to HIV prevention and treatment, SUD, and HCV care services post-release from the justice system. Addressing the gaps in care for persons involved in the justice system by linking to HIV, HCV, substance use, and other healthcare needs, as well as linkage to social support services is highly needed.

This project has two aims; the primary aim is to compare the effectiveness of two service delivery models, PN versus MHU, on participant length of time to initiation of PrEP or ART medication following release from custody. Additionally, this project will examine the continuation of PrEP and HIV care, including HIV viral suppression, PrEP and ART adherence, and HIV sexual and injection risk behaviors. The secondary aim of this study is to evaluate the feasibility and acceptability of these two models of care delivery. The study will also explore cost offsets and effectiveness. Furthermore, people who inject substances will be analyzed as a sub-study, focusing on factors associated with the effectiveness outcomes among this group of individuals. The impact of broader community health care, such as other health services accessed, expanded SUD services, and common barriers to service access across the community provider spectrum will also be explored. Overall, the study will provide highly informative practical knowledge to inform service delivery for this highly vulnerable population.

This project expands upon the *Ending the HIV Epidemic in the US* plan [10, 11] by not only addressing the need for diagnosing, treating, preventing, and predicting HIV outbreaks, but also highlighting the need for diagnosing, treating, and preventing HCV and substance use among some of the most vulnerable,- justice-involved persons who use drugs. Specifically, this project could provide valuable information for the provision of PrEP for those persons who use drugs involved in the justice system to prevent HIV including, upon FDA approval, injectable long-acting cabotegravir [67]. Results from this project will inform future planning and implementation of linkage to and receipt of HIV prevention and treatment services for persons who use opioids and/or stimulants who are at risk of acquiring or living with HIV released to the community from prison or jail.

## Data Availability

Not applicable.

## Abbreviations

ART: Antiretroviral therapy
CHW: Community health worker
DBS: Dried blood spot
DSMB: Data safety monitoring board
EPIS: Exploration, preparation, implementation, sustainment
GLMM: Generalized linear mixed model
HCV: Hepatitis C
HPTN: HIV prevention trials network
IRB: Institutional review board
NAS: Needs Assessment Survey
MHU: Mobile health unit
MEM: Mixed effect models
MOUD: Medication for opioid use disorder
OUD: Opioid use disorder
PHQ9: Patient health questionnaire 9
PLH: People living with HIV
PN: Patient navigator
PROMIS: Patient reported outcome measurement information system
PrEP: Preexposure prophylaxis
STI: Sexually transmitted infection
SUD: Substance use disorder
TCU: Texas Christian University
US: United States
VAS: Visual analog scale
VL: Viral load
VS: Viral suppression
